# Knocking out Analysis of the* CpxP* gene using Crispr/Cas9 in* Escherichia coli* MG1655

**DOI:** 10.1186/s13568-020-01099-z

**Published:** 2020-09-26

**Authors:** Xiaoliang He, Yuwen Ren, Wanli Meng, Xinran Yu, Xiaohui Zhou

**Affiliations:** School of Biological Science and Engineering, Hebei University of Science and Technology, No. 26 Yuxiang Street, Shijiazhuang, 050018 Hebei China

**Keywords:** Knocked out, *cpxP*, Crispr/Cas9, Homologous repair, *Escherichia coli*

## Abstract

Based on the analysis of *cpxP* genes among *Escherichia coli* strains, *cpxP* gene-targeting short guide RNA (sgRNA) was designed and inserted into the pGL3-MGP-RNA. The donor sequences (MG-HR) for homologous repair were designed and cloned by PCR. MG-HR and pGL3-MGP-RNA were transformed into *E. coli* MG1655 (pCas9). The *cpxP* gene expression cassette was amplified by PCR and subcloned into pBBR1MCS-2. Then the pBBR-*cpxP* was independently transformed into *E. coli* MG1655. The results of motility experiment suggest that *cpxP* gene had a significant effect on the movement ability of *E. coli* strain. The CpxP protein had a significant inhibition of bacterial activity. The lastest 81 CpxP proteins sequences were selected and analyzed by multi-sequence alignment and molecular cluster. The CpxP proteins were roughly divided into three categories. Our results suggest that the CpxP protein was involved in bacterial motility, infection and pathogenicity.

## Key points

The *E. coli* MG1655-*ΔcpxP* were successfully obtained.

The *cpxP* gene had a significant effect on the movement ability of *E. coli* strain.

The CpxP proteins had a significant inhibition of bacterial activity.

## Introduction

The Cpx system is one of the most common two-component regulatory systems in Gram-negative bacteria. It consists of the membrane-anchored sensor kinase CpxA, the cytosolic response regulator CpxR, and the peripheral spatial helper protein CpxP (Dong et al. 1993; Ruiz and Silhavy 2005). The CpxP proteins can inhibit activation of CpxA and are indispensable for the quality control system of P pili that are protein filaments expressed by uropathogenic *Escherichia coli* (Fällman et al. 2005; Nevesinjac and Raivio 2005). The structure of CpxP was interdigitated with two monomers like “left hands” forming a cap-shaped dimer. The structure revealed an antiparallel dimer of intertwined α-helices with a highly basic concave surface (Thede et al. 2011). The CpxP proteins inhibit the kinase CpxA through direct interaction between its concave polar surface and the negatively charged sensor domain on CpxA (Zhou et al. 2011).

The CRISPR-Cas system was used recently as efficient genome engineering technology in several prokaryotes and eukaryotes, including (but not limited to) *Escherichia coli* (Jiang et al. 2013), *Saccharomyces cerevisiae* (DiCarlo et al. 2013), yeast (DiCarlo et al. 2015), *Streptomyces spp*. (Cobb et al. 2014), higher plants (Shan et al. 2013), *Bombyx mori* (Wang et al. 2013), *Drosophila* (Yu et al. 2013), insects (Gantz and Bier 2015), *Anopheles stephensi* (Gantz et al. 2015), *Anopheles gambiae* (Hammond et al. 2016), mouse (Grunwald et al. 2019) and human cell lines (Cong et al. 2013; Mali et al. 2013; Zhang et al. 2013). The CRISPR/Cas9 system was also used to remove plasmid harbouring mcr-1 from *Escherichia coli* (Dong et al. 2019).

This study constructed an expression vector pGL3-MGP-RNA including gene-targeting short guide RNA and cloned a donor sequences (MG-HR) for homologous repair. The cell-envelope stress modulator *cpxP* gene of MG1655 strain was successfully knocked out by CRISPR-Cas9 system-based gene editing strategy. Likewise, the system can efficiently edit a large plasmid, target genes on the bacterial chromosome, or be adapted to introduce functional gene cargos alongside the gRNA cassette.

## Materials and methods

### Plasmid construction

The pCas9 and pGL3-U6-SgRNA-PGK-Puromycin Plasmid (Youbio, Hunan, China) was extracted from *E. coli* DH5α by Plasmid extraction kit (GenStar, Beijing, China). The pCas9 Plasmid was transfected into *E. coli* MG1655 (Tiangen, Beijing, China) to acquire *E. coli* MG1655 (pCas9). The transformed *E. coli* MG1655 was screened using Kanamycin (50 mg/L) that was added to the LB medium. Genomic DNA was extracted from *E. coli* MG1655 by Bacterial genome extraction kit (GenStar, Beijing, China).

The MG-HR-S and MG-HR-X were amplified by PCR using the genomic DNA as a template. The primers were designed with prime primer 5.0. The primers were synthesized by the Beijing Invitrogen Biotechnology Company. The primers TCTGGTGTGTCTGGCGAAGT and TGCTAATTCGTGGAGCTTATGCCAGCG TTGAGGCCATG were used to amplify MG-HR-S. The primers TAAGCTCCACGAATTAGCATCAGCAGATGCGAGATCTTAT and CTATGGC AAGGAAAACAGGGT were used to amplify MG-HR-X. MG-HR-S was connected to MG-HR-X by PCR to acquire a MG-HR.

The MGP-sgRNA and pGL3 were amplified by PCR using the pGL3-U6-SgRNA-PGK-Puromycin Plasmid as a template.

The primers CGGGATCCTTGACAGCTAGCTCAGTCCTAGGTATAATACTAG TTCAGGCGATAACTGGCATCCGTTTTAGAGCTAGAAAT and GGGGTACCGG AACCACGCCCAGAGCAG were used to amplify MGP-sgRNA.

The primers GGGGTACCGCTCACTGACTCGCTGCGCT and CGGGATCCGC TTAATGCGCCGCTACAGG were used to amplify pGL3.

The *cpxP* gene expression cassette comprising said promoter, a *cpxP* gene and said terminator was amplified by PCR using the genomic DNA as a template.

*cpxP*-F: CCCAAGCTTACGCGGTCTAATTCACTGCC 3′

*cpxP*-R: CGCGGATCCAGACAGGGATGGTGTCTATGGC 3′

The PCR was carried out by using the Gene Amp PCR system 9700 (Applied Biosystems). PCR products were confirmed on 1.0% agarose gels and recycled by the agarose gel extraction kit (Macherey–Nagel, Germany).

### Transformation of recombinant plasmid

Recycled PCR products of the MGP-sgRNA and pGL3 were digested by KpnI and BamHI (Thermo Fisher, USA),and then connected by T4 DNA Ligase (Thermo Fisher, USA) to acquire a pGL3-MGP-sgRNA.

400 μg pGL3-U6-sgRNA-PGK-puromycin, 400 μg pGL3-MGP-RNA, 400 μg pGL3-MGP-RNA and 1.6 μg MG-HR were separately transformed into *E. coli* MG1655 (pCas9). The transformed *E. coli* MG1655 (pCas9) were screened using Kanamycin (50 mg/L) and Ampicillin (100 mg/L) that were added to the LB medium.

Recycled PCR products of the *cpxP* gene expression cassette were digested by BamHI and HindIII (Thermo Fisher, USA), purified by the agarose gel extraction kit. The purified PCR products were directly subcloned into pBBR1MCS-2 (Youbio, Hunan, China) (No. pBBR-*cpxP*). Then the pBBR-*cpxP* was independently transformed into *E. coli* MG1655 to acquire the overexpression transformants and *E. coli* MG1655-*ΔcpxP* to acquire a revertant. The transformed *E. coli* MG1655 were screened using Kanamycin (50 mg/L).

### Motility experiment

*Escherichia coli* MG1655, overexpression transformants, the knocked out mutants and revertants were inoculumned on LB medium for overnight. Then the cultures (2 μl) were added to the LB semi-solid medium for overnight.

### Bacteriostatic experiment

200 μl ddH_2_O, 200 μl Kan (2.5 mg/ml) and 200 μl CpxP proteins (8 mg/ml) were independently added to the LB solid medium by 6 mm Oxford cup containing 100 μl *E. coli* MG1655 and *E. coli* K88 (10^5^ cfu/ml) for 16 h at 4 °C. Then they were kept at 37 °C for bacteriostatic experiment.

### Molecular clustering

To evaluate and analyze CpxP sequence resource preliminary, we had found 81 CpxP sequences from public database (http://www.ncbi.nlm.nih.gov/) and done multi-sequence alignment and molecular cluster by Clustal X and Treeview (He et al. 2015).

## Results

### Cloning of *cpxP* gene fragment

The *cpxP* gene fragments were cloned by PCR using MG-HR-S-F and MG-HR-S-R, MG-HR-X-F and MG-HR-X-R as primers. The results showed that the lengths of *cpxP* gene fragment were 424 bp and 377 bp respectively. The lengths of MG-HR-S and MG-HR-X were 782 bp (Additional file 1: Figure S1).

### Identification of pGL3-MGP-sgRNA plasmid

To confirm whether the MGP-sgRNA and pGL3 were connected by T4 DNA Ligase, the recombinant plasmids (pGL3-MGP-sgRNA) were digested with KpnI and BamHI. According to the electrophoresis, the recombinant plasmids (pGL3-MGP-sgRNA) were successfully obtained (Additional file 1: Figure S2).

### Identification of the knockout of *cpxP* gene

The knockout of *cpxP* gene was identified by PCR using MG-HR-S-F and MG-HR-X-R as primers, the genomic DNA of *E. coli* MG1655 (pCas9) as a template. According to the electrophoresis, the lengths of PCR products were 888 bp without knockout and 782 bp after knockout of *cpxP* gene (Additional file 1: Figure S3A). The PCR products were sent to the Beijing Invitrogen Biotechnology Company for sequencing. The sequences were done multi-sequence alignment by DNAstar (Additional file 1: Figure S3B). These results suggest that the *E. coli* MG1655-*ΔcpxP* were successfully obtained.

### Identification of pBBR-*cpxP* plasmid

PCR products of the *cpxP* gene expression cassette were digested by BamHI and HindIII, and cloned into pBBR1MCS-2. According to the electrophoresis, the recombinant plasmids (pBBR1MCS-2), the overexpression transformants and revertant were successfully obtained (Additional file 1: Figure S4).

### Motility experiment

**Fig. 1:**
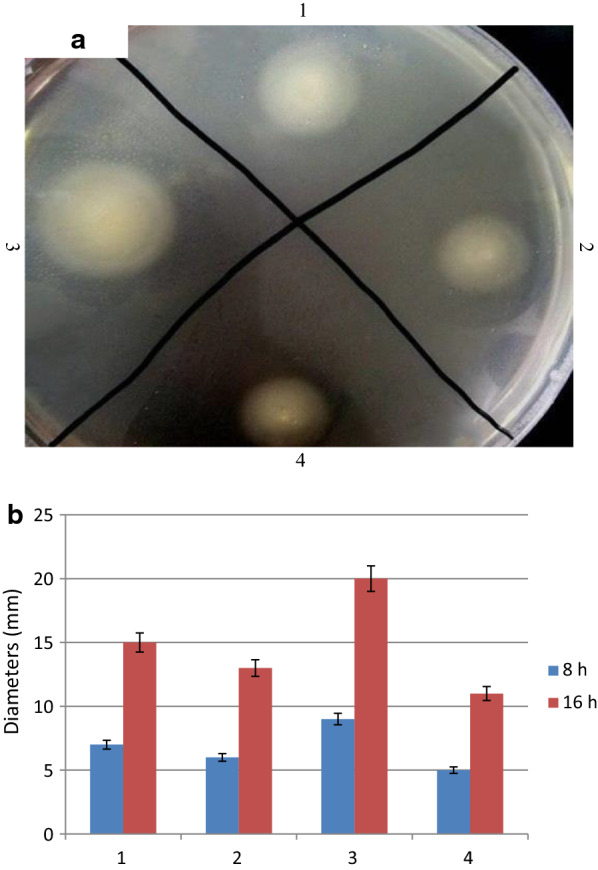
**a** Motility experiment of the strains. (1) Motility experiment of *E. coli* MG1655. (2) Motility experiment of the revertants. (3) Motility experiment of *E. coli* MG1655-*ΔcpxP.* (4) Motility experiment of the overexpression transformants. **b** Diameters of the strains in the LB semi-solid medium for 8 h and 16 h. (1) Diameters of *E. coli* MG1655. (2) Diameters of the revertants. (3) Diameters of *E. coli* MG1655-*ΔcpxP.* (4) Diameters of the overexpression transformants. Values are the mean ± SD (n = 5)

Many studies have confirmed that the motility of bacteria plays an important role in the pathogenicity of bacteria during the early interaction with the host. To investigate the effect of the *cpxP* gene on the motility of *E. coli*, the mobility of the strain was determined. The results showed that the diffusion diameters of *E. coli* MG1655-*ΔcpxP* is significantly greater than *E. coli* MG1655 (Student’s t-test, P < 0.05) (Fig. 1). The diffusion diameters of the overexpression transformants are significantly smaller than *E. coli* MG1655 (Student’s t-test, P < 0.05). These results suggest that *cpxP* gene had a significant effect on the movement ability of *E. coli* strain.

### Bacteriostatic experiment

**Fig. 2:**
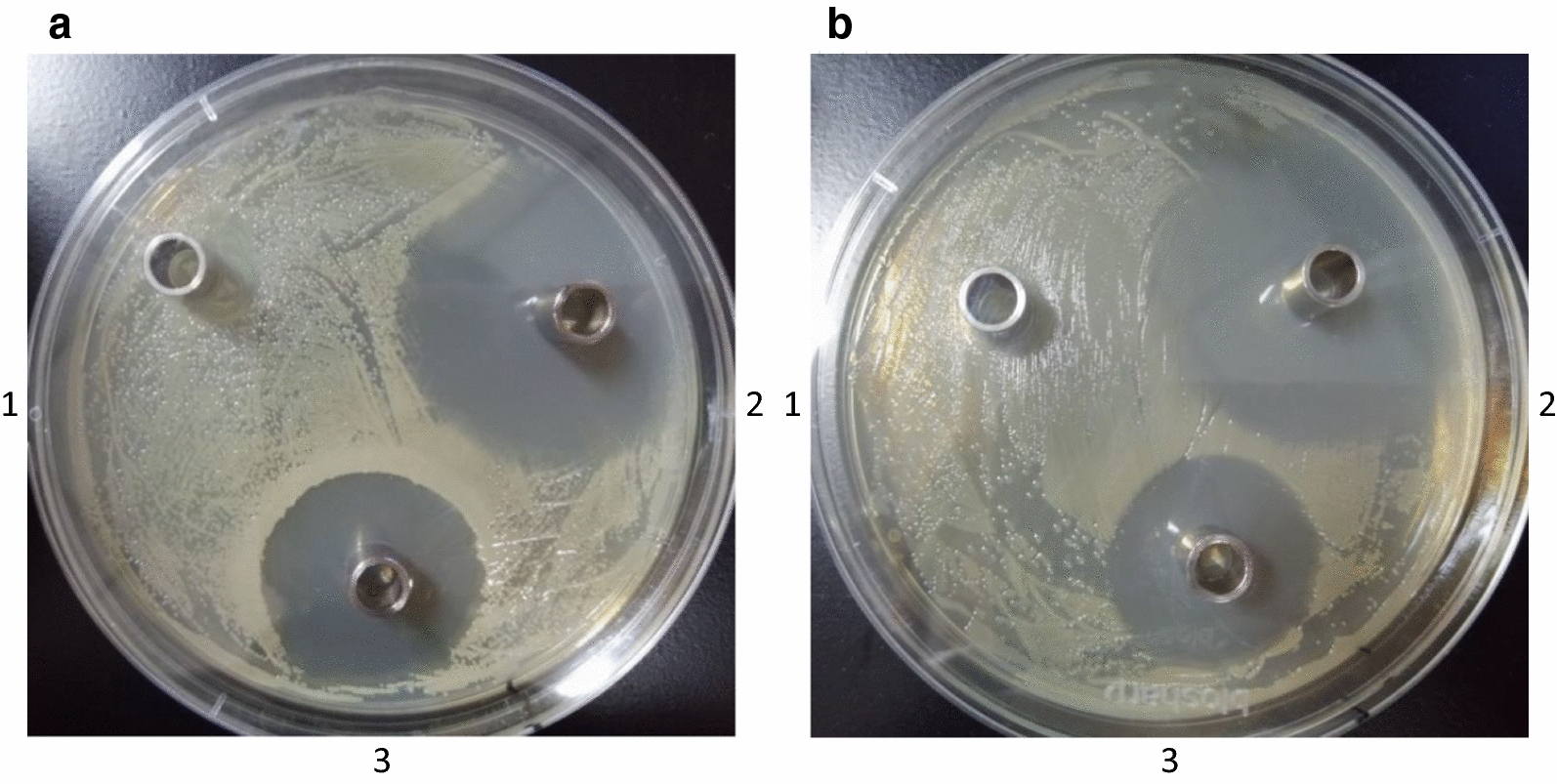
Antibacterial effect. **a** Antibacterial effect to *E. coli* K88. (1) 200 μl ddH_2_O. (2) 200 μl CpxP proteins. (3) 200 μl Kan (2.5 mg/ml). **b** Antibacterial effect to *E. coli* MG1655. (1) 200 μl ddH_2_O.0 (2) 200 μl CpxP proteins. (3) 200 μl Kan (2.5 mg/ml)

To investigate the affect of the CpxP proteins on the antibacterial, the diameters of inhibition zone were determined. The results (Fig. 2) showed that the inhibition effect of CpxP proteins was significantly greater than ddH_2_O (Student’s t-test, P < 0.01), and there was significant difference compared with Kan (Student’s t-test, P < 0.05) (Table 1). These results suggest that CpxP proteins had a significant inhibition of bacterial activity.

**Table 1:** Inhitory circle of CpxP after dispersed to two kinds of pathogen vobrio

	Inhibitory circle (mm)	
	*E. coli* K88	*E. coli* MG1655
ddH_2_O	0	0
CpxP proteins	27 ± 1.15	29 ± 1.25
Kan	37 ± 1.23	34 ± 1.19

Values are the mean ± SD (n = 4)

### Molecular clustering

**Fig. 3:**
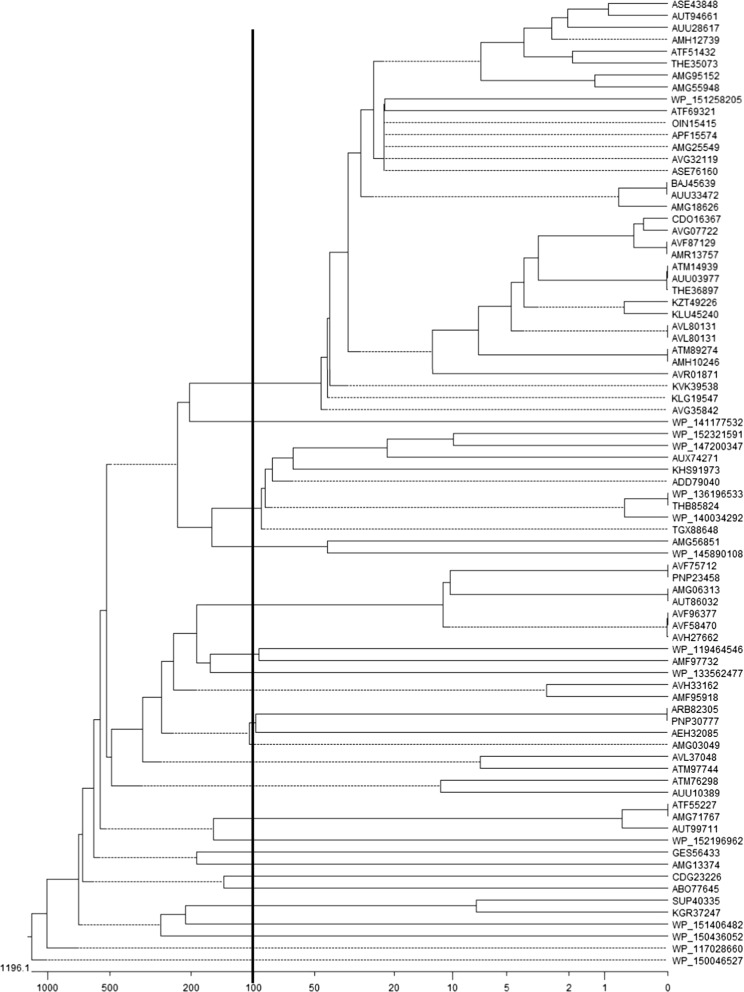
Molecular cluster of 81 CpxP protein sequences from 8 microbial species by Clustal X and Treeview

Multi-sequence alignment and molecular cluster was carried out for 81 CpxP proteins sequences. The results indicated that CpxP were roughly divided into three categories (Fig. 3). The protein sequence of the *E. coli* CpxP (BAJ45639) exhibited 100%, 100% and 89.2% homology with the *Shigella flexneri* (AUU33472), *Shigella sonnei* (AMG18626) and *Salmonella enterica* (AMG25549) homologous proteins, respectively. All the bacterial names corresponding to the gi numbers and sequences were showed (Additional file 1: Table S1).

## Discussion

The cell-envelope stress modulator *cpxP* (periplasmic protein) gene has been investigated for many years, but there are few studies on its function. The CpxP proteins can inhibit activation of CpxA and are indispensable for the quality control system of P pili.

In this report, the results showed that the *cpxP*-overexpression *E. coli* MG1655, the knocked out mutants *E. coli* MG1655-*ΔcpxP* and the revertants *E. coli* MG1655-*ΔcpxP* (pBBR-*cpxP*) were obtained. Deprivation of the *cpxP* gene resulted in significant enhancement in the mobility of *E. coli* strains. The overexpression of the *cpxP* gene also resulted in significant attenuation in the mobility of *E. coli* strains. The mobility of *E. coli* revertants strains was lower than *E. coli* MG1655. The mobility of bacteria had important pathological significance, moreover, and mainly played its role during the early stage of the infection (Mao and He 1998). In an experimental urinary tract infection of the mouse, colonization of the urinary bladder by isogenic strains of *Salmonella enterica* serovar Typhimurium was found to depend on the motility of the bacteria (Siitonen and Nurminen 1992). Our results suggest that the deprivation of the *cpxP* gene resulted in significant enhancement in the mobility and infection of *E. coli* strains.

Multi-sequence alignment and molecular cluster indicated that the CpxP proteins had a high homology at protein level with *Shigella flexneri*, *Shigella sonnei* and *Salmonella enteric* which are the main pathogenic bacteria in China. Our results suggest that the overexpressions of the *cpxP* gene may significantly reduce the pathogenicity of these bacteria.

## Supplementary information

**Additional file 1.** The electrophoregram and sequences of cpxP.

## Data Availability

Not applicable.
