# Supplementary material for: Knocking out Analysis of the CpxP gene using Crispr/Cas9 in Escherichia coli MG1655
Source: AMB Express. 2020 Sep 26;10:172. doi: 10.1186/s13568-020-01099-z (PMC7519928; doi:10.1186/s13568-020-01099-z)
Supplement: Supplementary file 1 — Additional file 1. The electrophoregram and sequences of cpxP. [file 13568_2020_1099_MOESM1_ESM.pdf]

Journal name: Applied Microbiology and Biotechnology

Title: Knocking out Analysis of the *CpxP* gene using Crispr/Cas9 in *Escherichia coli* MG1655

Authors' names: Xiaoliang He, Yuwen Ren, Wanli Meng, Xinran Yu, Xiaohui Zhou*

Authors' affiliations: School of Biological Science and Engineering, Hebei University of Science and Technology, Shijiazhang, Hebei, China

Corresponding author name: Xiaohui Zhou

Telephone: 86 311 81668487

Fax: 86 311 81668487

E-mail: zhouxh2003@aliyun.com

Address: School of Biological Science and Engineering, Hebei University of Science and Technology, NO.26 Yuxiang Street, Shijiazhang, Hebei, 050018, China

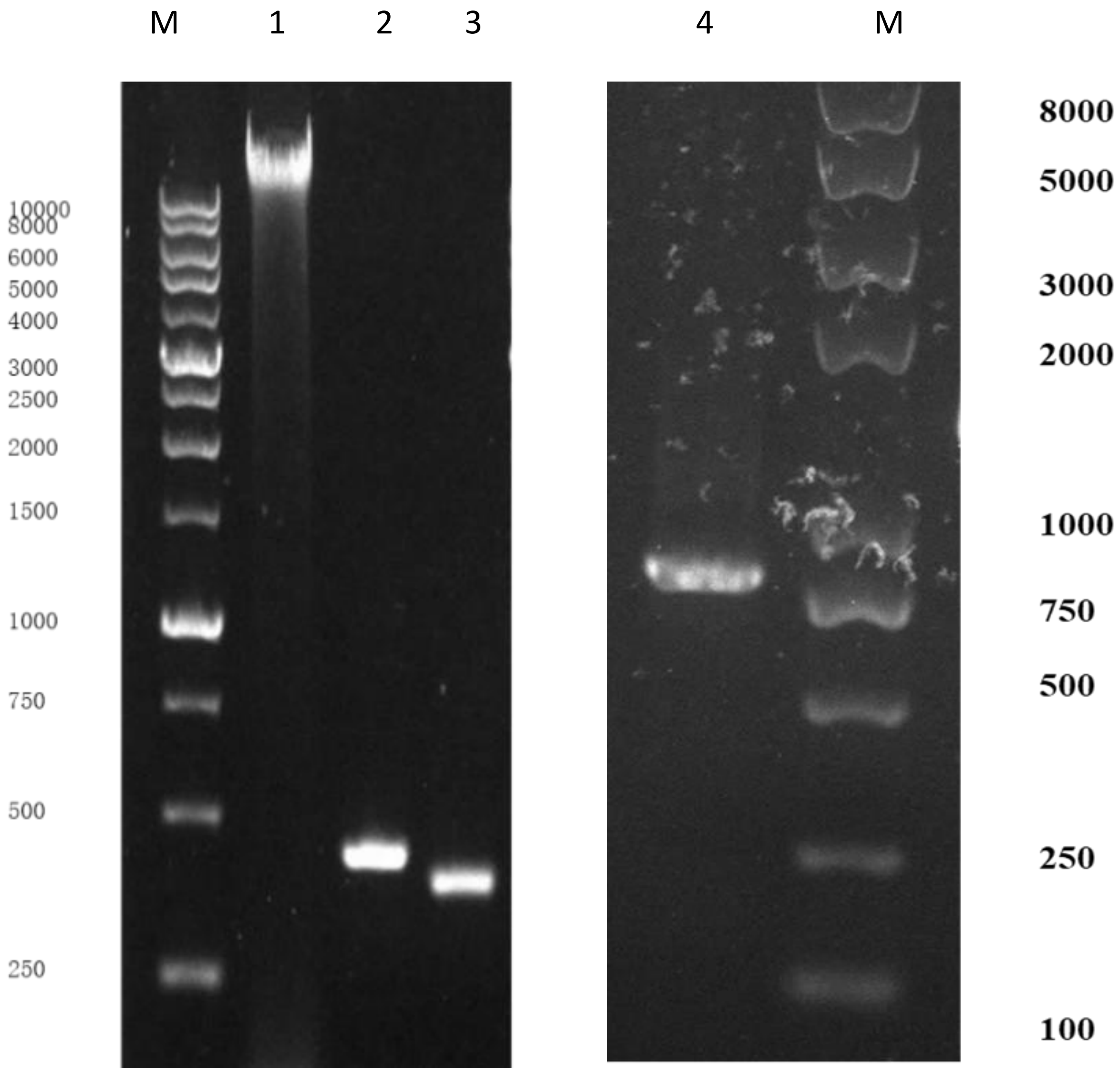

Figure S1 Cloning and identification of the *cpxP* gene fragments in *Escherichia coil* MG1655. (1) Genomic DNA of *Escherichia coil* MG1655. (2) PCR products MG-HR-S. (3) PCR products MG-HR-X. (4) MG-HR(MG-HR-S and MG-HR-X). M: marker.

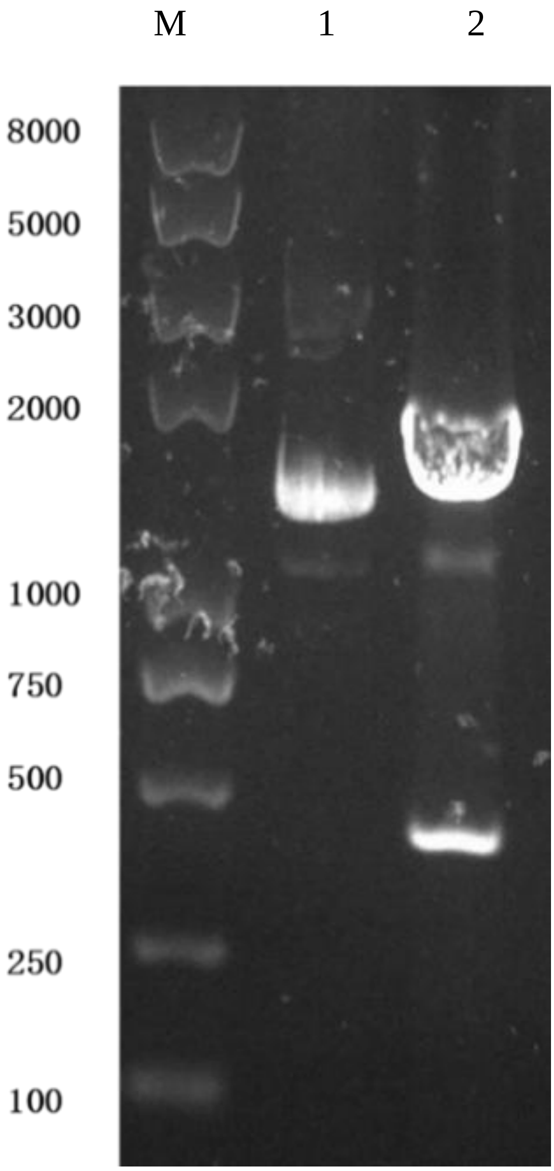

Figure S2 Construction and identify of pGL3-MGP-sgRNA plasmid. (1) pGL3-MGP-sgRNA plasmid. (2) Restriction enzyme identify with pGL3-MGP-sgRNA plasmid (*Kpn* I and *BamH* I). M: marker.

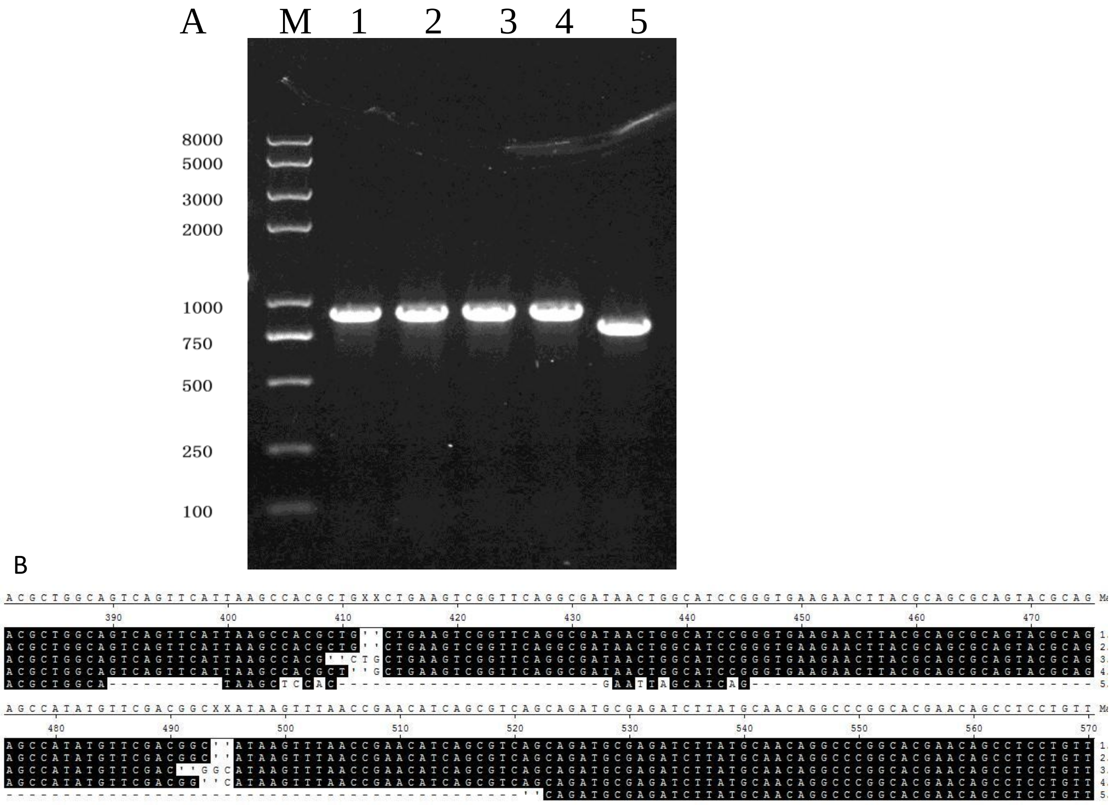

Figure S3 A. Identification of the knockout of *cpxP* gene. (1) PCR products of MG1655 (pCas9) with 400 μg pGL3-U6-sgRNA-PGK-puromycin. (2) PCR products of MG1655 (pCas9) with 400 μg pGL3-U6-sgRNA-PGK-puromycin. (3) PCR products of MG1655 (pCas9) with 400 μg pGL3-MGP-RNA. (4). PCR products of MG1655 (pCas9) with 400 μg pGL3-MGP-RNA. (5). PCR products of MG1655 (pCas9) with 400 μg pGL3-MGP-RNA and 1.6 μg MG-HR. M: marker. B. The sequences of PCR products. (1) The sequences of PCR products of MG1655 (pCas9) with 400 μg pGL3-U6-sgRNA-PGK-puromycin. (2) The sequences of PCR products of MG1655 (pCas9) with 400 μg pGL3-U6-sgRNA-PGK-puromycin. (3) The sequences of PCR products of MG1655 (pCas9) with 400 μg pGL3-MGP-RNA. (4). The sequences of PCR products of MG1655 (pCas9) with 400 μg pGL3-MGP-RNA. (5). The sequences of PCR products of MG1655 (pCas9) with 400 μg pGL3-MGP-RNA and 1.6 μg MG-HR.

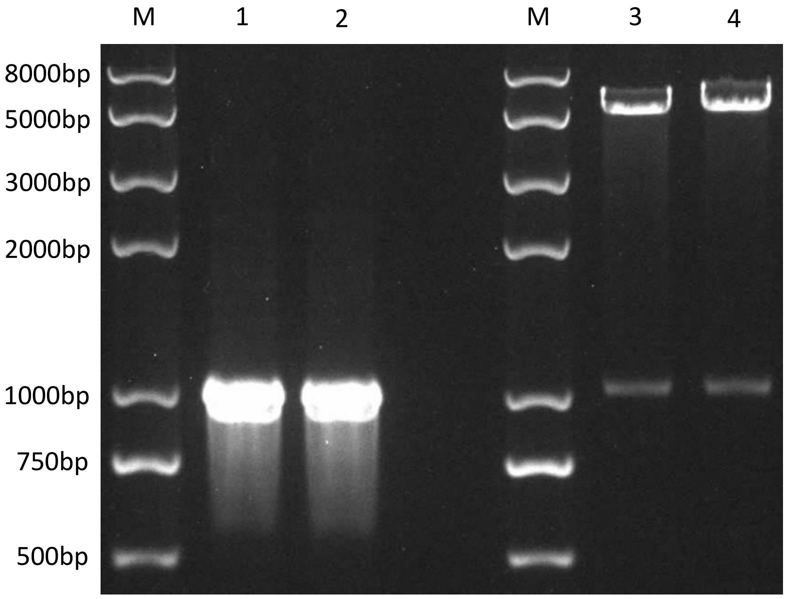

Figure S4 Construction and identify of pBBR-*cpxP* plasmid. (1) PCR products of the *cpxP* gene expression cassette. (2) Restriction enzyme identify with the *cpxP* gene expression cassette (*BamH* I and *Hind* III). (3) Restriction enzyme identify with pBBR-*cpxP* plasmid (*BamH* I and *Hind* III) of the overexpression transformants. (4) Restriction enzyme identify with pBBR-*cpxP* plasmid (*BamH* I and *Hind* III) of the revertant. M: marker.

Table S1

>CDO16367.1 cpxP [Klebsiella pneumoniae]
MRNVIAAVMASTLALSAYSQAAEVVTSVNWLPGDDGGQRGSQSHMFDGISLTEQQRQQLRDLMQRARHD
RLPVNVSEMETMHRLVTAENFDENAVRAQAEKMAQEQVARQVEMAKVRNQMYHLLTPEQQAVLNAKHQQ
RMDQLREVARMQKGSAMMLSSSSNTLQPQ
>ADD79040.1 CpxP [Pantoea ananatis LMG 20103]
MRKLTAVVIASAMVLCNASARAAEPMAPDDVHHGEFTTGSMTQNSQSHMFDGIELTEHQRQQMRDLMQQ
ARHDRPATNIDDIAAMHDLVTADNFNEAAIRSKAEAIARVQVEQQVEMARVRNQMYHLLTPEQQAALQKNY
ERRINSLRKLSNLQPASSLQPVSRTSSNQ
>BAJ45639.1 cpxP [Escherichia coli DH1]
MRIVTAAVMASTLAVSSLSHAAEVGSGDNWHPGEELTQRSTQSHMFDGISLTEHQRQQMRDLMQQARHEQ
PPVNVSELETMHRLVTAENFDENAVRAQAEKMANEQIARQVEMAKVRNQMYRLLTPEQQAVLNEKHQQR
MEQLRDVTQWQKSSSLKLLSSSNSRSQ
>CDG23226.1 CpxP [Xenorhabdus poinarii G6]
MRNIAILAVASMFVLETTETLANTADADHVPEAHSSPSCMQGDYKRNFSYYRGNQYNYSYIFGGIVLTEQ
QREQILRLAREQGGYEQPLADMQDAHFKLDDLLTEEDFDETEVRSLLEKIAEKHVLLGIEVARFNNQVYQ
LLTAEQKALLKKRKTSKCLTQNVN
>ABO77645.1 CpxP [Xenorhabdus nematophila]
MRNIAILALASMVVLRSTLALADTADTDDTPEAANPSPYCLSYEHKRDSGYYRSDEHNYNYSYVFGGITL
TEQQRQQMWDLVKKQHLHEQSIIDMRVERQKMYHLLIEREFDEAAVRLQLEKIAEKNIDLGVEIARIRNQ
MYQLLTPEQKERLYKRYEGQTAQEMH
>SUP40335.1 CpxP [Vibrio harveyi]
MKRYLLSTILLLSSANVFAEAAPTQPAAPKPFGLESITADQQAKIEEIQINLSSKLAGQQDPKAVQESAK
QFEKLVKASSFDEAKAKQLIQQAHAKQLDTQLAQLKAQHDIYNVLTAEQKSTLEKRQQEQLKKLEAMKQQ
QAQ
>KGR37247.1 CpxP [Vibrio campbellii]
MKRYLLSTILLLSSANVFAESAPAQPAAPKPFGLESITTEQQAKIEEIQIKLSSELAGNQDPKAVQESAK
QFEKLVKASSFDEVKATKLIKQAHAKQLDTQLAQLKAQHDIYNVLTAEQKATLEKRQQEQLKKFEALQKD
AQ
>AEH32085.1 CpxP [Vibrio anguillarum 775]
MMKMAKKIVLAAVILPLTLSTASVFAFGGKDQHKGPNDECGGFDRGMMQQLDLTSDQQAKLKGMREANRE
AMKGEHKGQRQAKMKAHHDKVQALVLAESFDAAAANELAKEMVDQQVAHRVKMLEKRHDMMSVLTPEQ
KAKLQTLQQDRMQKCMENGPKHMKKNS
>WP_136196533.1 cell-envelope stress modulator CpxP [Pantoea allii]
MRKLTAVVLASAMALSVASADAKDATTIDEMHHGGLPTGSMTQNPQSHMFDGIELTEEQRQQMRDLMQQ
ARHDRPVVHIDDIAALHELVTADQFNEAAIRQKAEVIARVQVEQQVEMARVQNQMFQLLTPAQQSTLQKNY
QRRLNELRQFSNLQSASSLQAVSSTSSNQ
>WP_133562477.1 CpxP family protein [Marinomonas communis]
MNLSKKLMMVTLALPLAFGTASSFAAGDQHERGGKGGRGHGEHQVCSGTAGLIYKLDLSDAQKEQLKELR
SVRHAQAKANAEKDIEQKRADRAQAHATMQKIVMADKFDTAAAKQFAGGMASKRAERNVMKMEAEHEM
FSVLTAAQKEQFLELQKTAGDDCKAKKKGKDGKRRHHEKAEQK
>WP_119464546.1 stress adaptor protein CpxP [Vibrio sp. PID23_8]

MKRLKSRTIEMLILPLVFASTSAIADSHEQEYDEKFHDKCGISMNLDRGMVHQLDLTDEQKAKLKSIREA
HKQEKKQTNVNRKVEQKERHQRMQAIVLESEFNHAKANGFAQEVAAIQAERSVQMMKNKHEMLSVLTTEQ
KAKFVQLQDDRQKECSNKRHQRNDSKMTE
>WP_117028660.1 stress adaptor protein CpxP, partial [Klebsiella pneumoniae]
SLTEHQRQQMRDLMQQARHEQPPVNVSELETMHRLVTAENFDENAVRAQAEKMANEQIARQVEMAKVRN
QMYRLLTPEQQAVLNEKHQQRMEQLRDVTQWQKSSSLKLLSSSNSRSQ
>GES56433.1 periplasmic repressor CpxP [Desulfuromonas sp. AOP6]
MKKRLIALTLLATALGGFVVAPTLALAADQNPAAETNQEERRCGKRGSCNGPFEGRMAAKLNLTSEQQAQ
IKAIVEAERERIAPLREQQREQRAQLQAAMKAQPFDENTVRQLAASQADARTEMIVHRARVQNQINAVLT
EEQREQAEQMRASKKDRRCNKGHRGFGFGFDQQNS
>WP_152321591.1 cell-envelope stress modulator CpxP [Erwinia endophytica]
MRKVTAVVVVPTLILSFSTAWATEVTTTDEMHQDNAVPRSMAQIPQSHMFDGISLTEQQRQQMRDLMQQA
RHERSPISISDLEQLHDMIIADKFDETAYRARLDKIAQAEVARQVEIAHVRNQMYHLLTPAQQDVLNQKH
QQRMDEMRKLASMPQASSLQAVSSTQQ
>WP_152196962.1 cell-envelope stress modulator CpxP [Rouxiella sp. S1S-2]
MCKVAAMIMASMLALSSSVALAESAKFMPADTLAHDGNMLDRRNTMFDGINLTEQQRQQMRDLMHQAR
RDSPQINLKQMETMHELVTAENFDQAAVRAQAEKIAQEQVDRQVEMARIRNLMFNLLTPQQKEILNQKHEQ
RMQVLAAQISGLQPTSTQKPVISTQ
>WP_151406482.1 cell-envelope stress modulator CpxP [Enterobacter hormaechei]
MTLRCFTPLTHVCSLNRRLSLVESRHERFWEQVMRKVTAAVMASTLAFSAFSRAAVAIISDNGSSAEGAT
QHSSQSHMFDGISLTEHQRQQMRDLMQRARHDQPLLMLAKWRQCIALSPQKILTKALYALRPKKWRRNRL
PAVEMAKVRNQMFHLLTPEQQAVLNTKHQQRMNQLREVARMQRSSDMTLFSSNSSTRSNQ
>WP_151258205.1 cell-envelope stress modulator CpxP [Salmonella enterica]
MRKVTAAVMASTLAFSFLSHAAEVVTSDNWHPGDGATQRSAQNHMFDGISLTEHQRQQMRDLMQQARHE
QPPVNVSEMETMHRLVTAEKFDESAVRAQAEKMAQEQVVTAEKFDESAVRAQAEKMAQEQVARQVEMAR
VRNQMYRLLTPEQQAVLNEKHQQRMEQLRDVAQWQKSSSLKLLSSSNSRSQ
>AVL37048.1 stress adaptor protein CpxP [Yersinia intermedia]
MRKVTKVTTLIMASMLVLGSQAAFAADKTGATDDWCHGDGTMMNKKDGRGHHNMFDGVNLTEQQRQQ
MRDLMRQSRQGQPRLDMADRDAMHKLVTADKFDEAAVRAQAEKMSKDQVERQVEMAKVRNQMFNLLTP
EQKAVLNQKHQQRIEKMQQAPAAQPSSAQK
>ATM97744.1 stress adaptor protein CpxP [Yersinia frederiksenii]
MRKVTKVSTLIMASMLVLGSQAAFAVDKTGPNDGWCHGDGMMMNKKDGRGHHNMFDGVNLTEQQRQQ
MRDLMRQSRQGQPRMDIADREAMHKLITADKFDEAAVRAQAEKMSKDQVDRQVEMAKVRNQMFNLLTP
EQKAALNQKHQQRIEKMRQVPAPAAQPASAQK
>ATM76298.1 stress adaptor protein CpxP [Serratia fonticola]
MVKVTAVVMASILALGSTAAFAADTTPETVQPPANDALLRALGQNHMFDGVRLTEQQRQQMRDLMRQARY
DLPGVNVDEVETMHKLVTADKFDEAAVQAQAEKMAQEQVKRQVEMARVRNQMYNLLTPEQKSVLDQKHQ
QRVQLMKQQISGLQQTSAQKLSMTE
>ASE76160.1 stress adaptor protein CpxP [Salmonella enterica]
MRKVTAAVMASTLAFSFLSHAAEVVTSDNWHPGDGATQRSAQNHMFDGISLTEHQRQQMRDLMQQARHE
QPPVNVSEMETMHRLVTAEKFDESAVRAQAEKMAQEQVARQVEMARVRNQMYRLLTPEQQAVLNEKHQQ
RMEQLRDVAQWQKSSSLKLLSSSNSRSQ
>ARB82305.1 stress adaptor protein CpxP [Vibrio cholerae]
MKLAKKMILAAAVLPLTLGTTAALAYGGHGWDKEGDGHCGDRGERGIWKQLDLTAEQQAQLKEMREAGRE

EMRANRGQSHDAMKALHAQERALVLAADFDQAAAENLAKQMVDQQVTHRVKMMEKRHQMMSILTAEQ
KAKLQTLQQEKMAECMQDGQHGKGKKHASQ
>AVH33162.1 stress adaptor protein CpxP [Vibrio fluvialis]
MKTAKKLMLAAVVLPIVLGSASALAAGGKNKGPDGEMCGPDGERGIFKQLNLTAEQHAKLRQMREEGREQ
MQQKRQAGPSEQMKAMRDKERALMLAPNFDKAQATELAKQMVDMQVERRVQMMEKRHQMLNVLTPE
QKTQFQNLQQERMAKCWENGPREGHHGDKGPKGQNMMPPAPPVGE
>AVG07722.1 stress adaptor protein CpxP [Klebsiella pneumoniae]
MRNVIAAVMASTLALSAYSQAAEVVTSVNWLPGDEGGQRGSQSHMFDGISLTEQQRQQLRDLMQRARHDR
LPVNVSEMETMHRLVTAENFDENAVRAQAEKMAQEQVARQVEMAKVRNQMYHLLTPEQQAVLNAKHQQR
MDQLREVARMQKGSAMMLSSSSNTLQPQ
>AVF96377.1 stress adaptor protein CpxP [Vibrio diabolicus]
MKSAKKLVLAAVVLPLTLGAASAFAYGGKNHHQGPRDECGMGMDRGIMRDLNLTDAQKDQLKSFREANRA
QMKGKYSENREARMAERQAHHAKMQSLLLADSFDEAQATALAKEMVERQTEHRVQMLERKHQMLSVLTPE
QKAEFVKLQNERMQECGDRMHKRMEKYRNN
>AVF75712.1 stress adaptor protein CpxP [Vibrio alginolyticus]
MKSAKKLVLAAVVLPLTLGAASAFAYGGKNHHKGPRDECGMGMDRGVMRQLDLTDAQKDQLKEMREANKA
EMKAKFADGKEARMAERQAHHAKVQSLLLADNFDEAQASELAKEMVERQTERRVQMLERKHQMLSVLTPE
QKAKFVELQNERMQECGDRMHKRMEKSRNN
>AVF58470.1 stress adaptor protein CpxP [Vibrio diabolicus]
MKSAKKLVLAAVVLPLTLGAASAFAYGGKNHHQGPRDECGMGMDRGIMRDLNLTDAQKDQLKSFREANRA
QMKGKYSENREARMAERQAHHAKMQSLLLADSFDEAQATALAKEMVERQTEHRVQMLERKHQMLSVLTPE
QKAEFVKLQNERMQECGDRMHKRMEKYRNN
>ATM89274.1 stress adaptor protein CpxP [Klebsiella aerogenes]
MRNVIAAVMASTLALSATSQAAEVVTGVNWLHGEEGAQRSGQSHMFDGISLTEQQRQQLRDLMQRARHDR
LPVNVSELETMHSLVTADKFDESAVRAQAEKMAQEQVARQVEMAKVRNQMYHLLTPEQQAVLNAKHQQR
MDQLREVARMQKGSAMMLSSSSSTVQPK
>ATM14939.1 stress adaptor protein CpxP [Raoultella planticola]
MRNVIAAVMASTLALSAYSQAAEVVTSVNWLPGDDGAQRTSQSHMFDGISLTEQQRQQLRDLMQRARHDR
QPINVSEMETMHRLVTAENFDENAVRAQANKMAQEQVTRQIEMAKVRNQMYHLLTPEQQAVLNAKHQQR
MDQLREVARMQKGSATMLSSSSNTVQPQ
>ATF69321.1 stress adaptor protein CpxP [Salmonella enterica subsp. enterica serovar Saintpaul]
MRKVTAAVMASTLAFSFLSHAAEVVTSDNWHPGDGATQRSAQNHMFDGISLTEHQRQQMRDLMQQARHE
QPPVNVSEMETMHRLVTAEKFDESAVRAQAEKMAQEQVARQVEMARVRNQMYRLLTPEQQAVLNEKHQQ
RMEQLRDMAQWQKSSSLKLLSSSNSRSQ
>ATF55227.1 stress adaptor protein CpxP [Morganella morganii]
MGKIATITLASMFVMQSAPGLAQDSESDSCVTPVQSHSQYKGITTSGGDGYTSMLTGIRLTEEQRMQLRD
LMHNYRDQLRNVRNLAEDDIALYELVKAEKFDETAVRNQLEKEMRKRLDYQVEMIRVHHQMYQLLNPEQK
MQLDANFEPESIHTSSASSAQNMPE
>ATF51432.1 stress adaptor protein CpxP [Citrobacter werkmanii]
MGKVTAAVMASTLALSTFSHAAEVVTSDHWHLGESSSQRNAQSHMFDGISLTEHQRQQMRDLMQQARHE
QPPVNVSEMETMHRLVTAENFDESAVRAQAEKMAQQQVARQVEIAKVRNQMYRLLTPEQQAVLNEKHEQR
MEQLRDVAQWQKSSSLNLLSSSNSRSQ
>AMG56851.1 stress adaptor protein CpxP [Pantoea vagans]
MRKLTAVVLASAMALSVASAGAKDATTIDEMHHDGGLPTGSMTQNPQSHMFDGIELTEEQRQQMRDLMQ

QARHERPVVHIDDIAALHDLVTADQFNEAAIRVKAEVIARVQVEQQVEMARVQNQMFQLLTPDQQATLQKN
YQRRLNELRQFSNLQSASSLQAVSSTSSNQ
>AMG13374.1 stress adaptor protein CpxP [Vibrio vulnificus]
MSKELIMKLAKKMVLAAVVLPLTLGTASAFAFGGGKGHHKGPDGECGMGMERGMMRQLDLTDAQKEQLDA
MRGSNRAQMKEMHQGNFAANQAERQAQHAKVQALLLADNFDQATANELAKQMAEKQAERRVKMLEKQ
HQMLSILTPEQKAKFVELQNERMQECGDKMQKRMEKHAKN
>AMG03049.1 stress adaptor protein CpxP [Vibrio mimicus]
MKLAKKMTLAAAILPLTLGTTAAFAYGGHGWDKEGDGPCGGHGERGIWKQLDLTAEQQTQLKEMRDANRE
EMRANRGQNRDAMKALHTQERALVLAADFDQAAAENLAKQMVDQQVAHRVKMMEKRHQMMSILTAEQ
KTKLQSLQQAQMDRCMMDGEHGKGKPRHQ
>AMF97732.1 stress adaptor protein CpxP [Vibrio harveyi]
MKTAKKLVLAAVVLPLTLGTASAFAFGGKDHKGHRGECGMGMDRGIMRQLDLTDAQKDQLKEMREANKAE
MKAKFADGKEARMAERQAHHEKVQALLLADNFDAAAANDLAKEMVEKQTERRVKMMEKKHQMLSVLTPE
QKTKFVELQKERQQKCGEKMQKRMEKHHNS
>AMF95918.1 stress adaptor protein CpxP [Vibrio fluvialis]
MKTAKKLMLAAVVLPIVLGSASALAAGGKNKGPDGEMCGPDGERGIFKQLNLTAEQHAKLRQMREEGREQ
MQQKRQAGPSEQMKAMRDKERALMLAPNFDKAQATELAKQMVDMQVERRVQMMEKRHQMLSVLTPEQ
KTQFQNLQQERMAKCWENGPREGHHGGKGSKGQNMMPPAPPAGE
>WP_150436052.1 cell-envelope stress modulator CpxP [Brenneria sp. L3-3HA]
MRQVSALSLVSLLVLGSSAAASETDNASKGIWSHDETATVTVSGHQGMFDGVRLTELQRQQMRDLMQLAR
QELPELNTNDVEVMHRLIIAEKFDEAAVRAQAEKMAQRQVVRQVEMAKVRNQMYNLLTSEQKQILAQKHQ
QRMESMRQQMDRVNQASARKQ
>AVR01871.1 stress adaptor protein CpxP [Pluralibacter gergoviae]
MRNVFAAVMASTLALSAHSQAAEVVNSVNWHPNEGVAQTSSQGHMFDGISLTEHQRQQMRDLMQQAM
RKQPPVNVSEIETMHKLVTAEKFDETAVRAQAEKMAQEQVARQVEMAKVRNQMYHLLTPEQQAVLNQKHQ
QRMDQLREVARMQQGAMPMLYSSNRSHQ
>AVL80131.1 stress adaptor protein CpxP [Klebsiella oxytoca]
MRNVIAAVMASTLALSAYSQAAEVVTSVNWLPGDEGVQRSSQSHMFDGISLTEHQRQQLRDLMQRARHER
PPVNVSEMETMHRLVTAENFDENAVRAQADKMAQEQVARQVEMAKVRNQMYHLLTPEQQAVLNAKHQQ
RMNQLREVARMQKGSAMMLSSSSNTLQPQ
>AVL80131.1 stress adaptor protein CpxP [Klebsiella oxytoca]
MRNVIAAVMASTLALSAYSQAAEVVTSVNWLPGDEGVQRSSQSHMFDGISLTEHQRQQLRDLMQRARHER
PPVNVSEMETMHRLVTAENFDENAVRAQADKMAQEQVARQVEMAKVRNQMYHLLTPEQQAVLNAKHQQ
RMNQLREVARMQKGSAMMLSSSSNTLQPQ
>ASE43848.1 stress adaptor protein CpxP [Citrobacter braakii]
MGKVTAAVMASTLALSTLSHAAEVVTGDHWHLGEGSAQRSVQSHMFDGISLTEHQRQQMRDLMQQARHE
QPPVNVSEMETMHRLVTAEKFDERAVRAQAERMAQEQVARQVEIAKVRNQMYRLLTPEQQAVLNERHEQR
MEQLRDVAHWKKSSSLNLLSSSNSRSQ
>AMH12739.1 stress adaptor protein CpxP [Citrobacter sp. FDAARGOS_156]
MGKVTAAVMASTLALSTLSHAAEVVTGDHWHLGEGSSQRSVQSHMFDGISLTEHQRQQMRDLMQQARHE
QPPVNVSEMETMHRLVTAEKFDESAVRAQAEKMAQEQVARQVEIARVRNQMYRLLTPEQQAVLNEKHEQR
MEQLRDVAHWKQSSSLNLLSSSNSRSQ
>AMH10246.1 stress adaptor protein CpxP [Klebsiella aerogenes]
MRNVIAAVMASTLALSATSQAAEVVTGVNWLHGEEGAQRSGQSHMFDGISLTEQQRQQLRDLMQRARHDR

LPVNVSELETMHSLVTADKFDESAVRAQAEKMAQEQVARQVEMAKVRNQMYHLLTPEQQAVLNAKHQQR
MDQLREVARMQKGSAMMLSSSSSTVQPK
>AMG95152.1 stress adaptor protein CpxP [Citrobacter amalonaticus]
MRKVTAAVMASTLALSTVSHAAEVVTGDNWHPGESPAPRTVQSHMFDGISLTEHQRQQMRDLMQQARHE
QAPVNVSEMETMHRLITAENFDETAVRAQAEKMAQAQVSRQVEMARVRNQMYRLLTPEQQAVLNEKHQQ
RMEQLRDVTQWQKSSSLKLLSSSNSRSQ
>AMG71767.1 stress adaptor protein CpxP [Morganella morganii]
MGKIATITLASMFVMQSAPGLAQDSESDSCVTPVQSHSQYKGITTSGGDGYTSMLTGIRLTEEQRMQLRD
LMHNYRDQLRNVRNLAEDDIALYELVKAEKFDETAVRNQLEKEMRKRLDYQVEMIRVHHQMYQLLNPEQK
MQLDANFEPESIHTSSASSAQNMPE
>AMG55948.1 stress adaptor protein CpxP [Citrobacter amalonaticus]
MRKVTAAVMASTLALSTVSHAAEVVTGDNWHPGESSAPRTVQSHMFDGISLTEHQRQQMRDLMQQARHE
QPPVNVSEMETMHRLITAENFDETAVRAQAEKMAQAQVARQVEMARVRNQMYRLLTPEQQAVLNEKHQQ
RMEQLRDVTHWQKSSSLKLLSSSNSRSQ
>AMG18626.1 periplasmic protein CpxP [Shigella sonnei]
MRIVTAAVMASTLAVSSLSHAAEVGSGDNWHPGEELTQRSTQSHMFDGISLTEHQRQQMRDLMQQARHEQ
PPVNVSELETMHRLVTAENFDENAVRAQAEKMANEQIARQVEMAKVRNQMYRLLTPEQQAVLNEKHQQR
M
>WP_150046527.1 cell-envelope stress modulator CpxP, partial [Klebsiella pneumoniae]
HMFDGISLTEQQRQQLRDLMQRARHDRLPVNVSEMETMHRLVTAENFDENAVRAQAEKMAQEQVARQVE
MAKVRNQMYHLLTPEQQAVLNAKHQQRMDQLREVARMQKGSAMMLSSSSNTLQPQ
>AVH27662.1 stress adaptor protein CpxP [Vibrio diabolicus]
MKSAKKLVLAAVVLPLTLGAASAFAYGGKNHHQGPRDECGMGMDRGIMRDLNLTDAQKDQLKSFREANRA
QMKGKYSENREARMAERQAHHAKMQSLLLADSFDEAQATALAKEMVERQTEHRVQMLERKHQMLSVLTPE
QKAEFVKLQNERMQECGDRMHKRMEKYRNN
>AVG32119.1 stress adaptor protein CpxP [Salmonella enterica subsp. enterica serovar Heidelberg]
MRKVTAAVMASTLAFSFLSHAAEVVTSDNWHPGDGATQRSAQNHMFDGISLTEHQRQQMRDLMQQARHE
QPPVNVSEMETMHRLVTAEKFDESAVRAQAEKMAQEQVARQVEMARVRNQMYRLLTPEQQAVLNEKHQQ
RMEQLRDVAQWQKSSSLKLLSSSNSRSQ
>AVF87129.1 stress adaptor protein CpxP [Klebsiella quasipneumoniae]
MRNVIAAVMASTLALSAISQAAEVVTSVNWLPGDEGGQRGSQSHMFDGISLTEQQRQQLRDLMQRARHDR
LPVNVSEMETMHRLVTAENFDENAVRAQAEKMAQEQVARQVEMAKVRNQMYHLLTPEQQAVLNAKHQQR
MDQLREVARMQKGSAMMLSSSSNTLQPQ
>AMG25549.1 stress adaptor protein CpxP [Salmonella enterica]
MRKVTAAVMASTLAFSFLSHAAEVVTSDNWHPGDGATQRSAQNHMFDGISLTEHQRQQMRDLMQQARHE
QPPVNVSEMETMHRLVTAEKFDESAVRAQAEKMAQEQVARQVEMARVRNQMYRLLTPEQQAVLNEKHQQ
RMEQLRDVAQWQKSSSLKLLSSSNSRSQ
>AMG06313.1 stress adaptor protein CpxP [Vibrio parahaemolyticus]
MKSAKKLVLAAVVLPLTLGTASAFAFGGKDHHKGPRDECGMGMDRGIMRDLNLTDAQKDQLQSFRDANRA
EMKGKYSQNREARMAERQAHHAKMQSLLLADTFDEAQATALAKEMVERQTEHRVKMLERKHQMLSVLTPE
QKAEFVKLQNERMQECGDQMQQRMGKHRNN
>AVG35842.1 stress adaptor protein CpxP [Enterobacter cloacae complex sp.]
MRKVTAAVMASTLAFSAFSQAAEAIISDNSPLQEGATQNSSQSHMFDGISLTEHQRQQMRDLMQRARHDQ

PPVNVSEMETMHRLVTAENFDESAVRAQAEKMAQEQVARQVEMAKVRNQMFHLLTPEQQAVLNTKHQQR
MDQLREVARMQRSSETSFFSSNSSTRSNQ
>AUU33472.1 periplasmic protein CpxP [Shigella flexneri]
MRIVTAAVMASTLAVSSLSHAAEVGSGDNWHPGEELTQRSTQSHMFDGISLTEHQRQQMRDLMQQARHEQ
PPVNVSELETMHRLVTAENFDENAVRAQAEKMANEQIARQVEMAKVRNQMYRLLTPEQQAVLNEKHQQR
MEQLRDVTQWQKSSSLKLLSSSNSRSQ
>AUU28617.1 stress adaptor protein CpxP [Citrobacter freundii]
MGKVTAAVMASTLALSTFSHAAEVVTGDHWHLGEGSSQRSVQSHMFDGISLTEHQRQQMRDLMQQARHE
QPPVNVSEMETMHRLVTAEKFDESAVRAQAERMAQEQVARQVEIARIRNQMYRLLTPEQQAVLNEKHEQR
MVQLRDVAHWKKSSSLNLLSSSNSRSQEQLRDVTQWQKSSSLKLLSSSNSRSQ
>AUU03977.1 stress adaptor protein CpxP [Raoultella planticola]
MRNVIAAVMASTLALSAYSQAAEVVTSVNWLPGDDGAQRTSQSHMFDGISLTEQQRQQLRDLMQRARHDR
QPINVSEMETMHRLVTAENFDENAVRAQANKMAQEQVTRQIEMAKVRNQMYHLLTPEQQAVLNAKHQQR
MDQLREVARMQKGSATMLSSSSNTVQPQ
>AUT86032.1 stress adaptor protein CpxP [Vibrio parahaemolyticus]
MKSAKKLVLAAVVLPLTLGTASAFAFGGKDHHKGPRDECGMGMDRGIMRDLNLTDAQKDQLQSFRDANRA
EMKGKYSQNREARMAERQAHHAKMQSLLLADTFDEAQATALAKEMVERQTEHRVKMLERKHQMLSVLTPE
QKAEFVKLQNERMQECGDQMQQRMGKHRNN
>AUU10389.1 stress adaptor protein CpxP [Serratia marcescens]
MRKVTALVMASLLAIGSTAAFAADTIPDTAQPPGNDAMTRIPGQHHMFDGVSLSEQQRQQMRDLMRQARH
DLPGVNVAEMEAMHKLVTAEKFDEAAVYAQAEKMAQQQVKRQVEMARVRNQMYNLLTPEQKSVLDQKHQ
QRMQQMEQQISGLQQASAQK
>AUT94661.1 stress adaptor protein CpxP [Citrobacter freundii]
MGKVTAAVMASTLAFSTLSHAAEVVTGDHWHLGEGSAQRSVQSHMFDGISLTEHQRQQMRDLMQQARHE
QPPVNVSEMETMHRLVTAEKFDESAVRAQAERMAQEQVARQVEIAKVRNQMYRLLTPEQQAVLNEKHEQR
MEQLRDVAHWKKSSSLNLLSSSNSRSQ
>AUT99711.1 stress adaptor protein CpxP [Morganella morganii]
MGKIATITLASMFVMQSAPGLAQDSESDGCVTPVQSHSQYKGITTSGGDGYTSMLTGIRLTEEQRMQLRD
LMHNYRDQLRNVRNLAEDDIALYELVKAEKFDETAVRNQLEKEMRKRLDYQVEMIRVHHQMYQLLNPEQK
MQLDANFEPESIHTSSASSAQNMPE
>WP_147200347.1 cell-envelope stress modulator CpxP [Pantoea sp. CCBC3-3-1]
MRKVTAVVVVPALILSFSAAWAAEVTTNDEMHQDGAALRSMTQIPQSHMFDGINLTEQQRQQMRDLMQQ
ARHERSSISINDLEQLHEMIIADKFNETDYKARLDRIAKEEVTRQVEMARVRNQMYHLLTPAQQDVLKQKH
QQRMSEMRKLAQMQQAVSLQAVSSPESKQ
>WP_145890108.1 cell-envelope stress modulator CpxP [Pantoea dispersa]
MRYLTAVVIASAMVLSQASAEAADTTTIDEMHQNGGLTSGSMTQNPQSHMFDGIELTEQQRQQMRDLMQ
QARHERPVVSVQDIETLHDLETADQFNENAVRQQAEKQAKAQVELQVEMARVRNQMYHLLSPSQQATLQK
NFERRLKEARRVAGLQPSSPLHAVSSTSSNQ
>APF15574.1 stress adaptor protein CpxP [Salmonella enterica subsp. enterica serovar Typhimurium]
MRKVTAAVMASTLAFSFLSHAAEVVTSDNWHPGDGATQRSAQNHMFDGISLTEHQRQQMRDLMQQARHE
QPPVNVSEMETMHRLVTAEKFDESAVRAQAEKMAQEQVARQVEMARVRNQMYRLLTPEQQAVLNEKHQQ
RMEQLRDVAQWQKSSSLKLLSSSNSRSQ
>AMR13757.1 stress adaptor protein CpxP [Klebsiella quasipneumoniae]

MRNVIAAVMASTLALSAISQAAEVVTSVNWLPGDEGGQRGSQSHMFDGISLTEQQRQQLRDLMQRARHDR
LPVNVSEMETMHRLVTAENFDENAVRAQAEKMAQEQVARQVEMAKVRNQMYHLLTPEQQAVLNAKHQQR
MDQLREVARMQKGSAMMLSSSSNTLQPQ
>AUX74271.1 stress adaptor protein CpxP [Erwinia pyrifoliae]
MRKVTAVVVVPALIITFFVAWSANAATTGEMHQDDGTNRTLRQVPQSNMFDGISLTEQQRQEMRDLMQQA
RYDRSPISISDLDQLHELIIADKFDKAAYEAQAKKIAHAEVARQVEMGRVRNQMYHLLTPQQQSILQQKH
QQRLGELRRLTNMQLSSPLQAASSTDSTP
>KZT49226.1 repressor CpxP [Klebsiella michiganensis M5al]
MRNVIAAVMASTLALSAYSQAAEVVTSVNWLPGDESVQRSSQSHMFDGISLTEQQRQQLRDLMQRARHDR
PPVNVSEMETMHRLVTAENFDENAVRAQADKMAQEQVARQVEMAKVRNQMYHLLTPEQQAVLNAKHQQ
RMDQLREVARMQKGSAMMLSSSSNTLQPQ
>KVK39538.1 repressor CpxP [Enterobacter chengduensis]
MRKVTAAVMASTLAFSAFSQAAVAINGDNGPSPEGATQLSSQSHMFDGISLTEHQRQQMRDLMQRARHDQ
PPVNVSEMETMHRLVTAENFDESAVRAQAEKMAQEQVARQVEMAKVRNQMFHLLTPEQQAVLNTRHQQR
MDQLREVARMQRSSEATFFSSNSSTRSNQ
>KLU45240.1 periplasmic repressor CpxP [Klebsiella michiganensis]
MRNVIAAVMASTLALSAYSQAAEVVTSVNWLPGDEGVQRSSQSHMFDGISLTEHQRQQLRDLMQRARHDR
PPVNVSEMETMHRLVTAENFDENAVRAQADKMAQEQVARQVEMAKVRNQMYHLLTPEQQAVLNAKHQQ
RMDQLREVARMQKGSAMMLSSSSNTLQPQ
>KLG19547.1 periplasmic repressor CpxP [Enterobacter roggenkampii]
MRKVTAAVMASTLAFSAFSQAAVAIISDNGSSQEDTTQHSSQSHMFDGISLTEHQRQQMRDLMQRARHDQ
PPVNVSEMETMHRLVTAENFDESAVRAQAEKMAQEQVARQVEMAKVRNQMFHLLTPEQQAVLNTKHQQR
MDQLREVARMQRSSETSFFSSNSSTRSNQ
>PNP30777.1 stress adaptor protein CpxP [Vibrio cholerae]
MKLAKKMILAAAVLPLTLGTTAALAYGGHGWDKEGDGHCGDRGERGIWKQLDLTAEQQAQLKEMREAGRE
EMRANRGQSHDAMKALHAQERALVLAADFDQAAAENLAKQMVDQQVTHRVKMMEKRHQMMSILTAEQ
KAKLQTLQQEKMAECMQDGQHGKGKKHASQ
>PNP23458.1 stress adaptor protein CpxP [Vibrio alginolyticus]
MKSAKKLVLAAVVLPLTLGAASAFAYGGKNHHKGPRDECGMGMDRGVMRQLDLTDAQKDQLKEMREANKA
EMKAKFADGKEARMAERQAHHAKVQSLLLADNFDEAQASELAKEMVERQTERRVQMLERKHQMLSVLTPE
QKAKFVELQNERMQECGDRMHKRMEKSRNN
>WP_140034292.1 cell-envelope stress modulator CpxP [Pantoea vagans]
MRKLTAVVLASVMALSVASADAKDATTIDEMHHGGLPTGSMTQNPQSHMFDGIELTEEQRQQMRDLMQQ
ARHDRPVVHIDDIAALHELVTADQFNEAAIRQKAEVIARVQVEQQVEMARVQNQMFQLLTPAQQSALQKNY
QRRLNELRQFSNLQSASSLQAVSSTSSNQ
>WP_141177532.1 cell-envelope stress modulator CpxP [Mixta sp. BIT-26]
MRKVTAAVLASASVVLSYSSAWAADVNTVSVMQQPGEAMFGSIAQNLQSHMFDGIKLSEQQRQQMRDLM
QQTQYARPLVNVKEIETLHNLIIADEFNEAAVRAQAEKLAQAQVVRQVEMSRIRNQMYHLLTPQQQAVLQT
RHEQRMNELRRLTNQQQVPSLHEASSTGSNQ
>KHS91973.1 periplasmic stress adaptor protein CpxP [Pectobacterium polaris]
MQRFATLSLASLLMLGTFTAFAAESGDASASGWHIDDSATKGAPGQQGMFDGVRLTEQQRQQMRDLMHQS
RQDKPAFNAEDVKAMHQLVTAETFDEAAVRAQITRMMSVQLERQIQMTRVRNQMYNLLTPAQKEILELKH
KQRMKEMQQQISMFNQMAAPSPGMTSQTETNNPE
>OIN15415.1 stress adaptor protein CpxP [Salmonella enterica subsp. enterica]

MRKVTAAVMASTLAFSFLSHAAEVVTSDNWHPGDGATQRSAQNHMFDGISLTEHQRQQMRDLMQQARHE
QPPVNVSEMETMHRLVTAEKFDESAVRAQAEKMAQEQVARQVEMARVRNQMYRLLTPEQQAVLNEKHQQ
RMEQLRDVAQWQKSSSLKLLSSSNSRSQ
>THE36897.1 stress adaptor protein CpxP [Raoultella ornithinolytica]
MRNVIAAVMASTLALSAYSQAAEVVTSVNWLPGDDGAQRTSQSHMFDGISLTEQQRQQLRDLMQRARHDR
QPINVSEMETMHRLVTAENFDENAVRAQANKMAQEQVTRQIEMAKVRNQMYHLLTPEQQAVLNAKHQQR
MDQLREVARMQKGSATMLSSSSNTVQPQ
>THE35073.1 stress adaptor protein CpxP [Citrobacter murliniae]
MGKVTAAVMASTLALSTLSHAAEVVTGDHWHLGESGSQRNAQSHMFDGISLTEHQRQQMRDLMQQARHE
QPPVNVSEMETMHRLVTAEKFDESAVRAQAEKMAQEQVARQVEIAKVRNQMYRLLTPEQQAVLNEKHEQR
MEQLRDMAQWQKSSSLNLLSSSNSRSQ
>THB85824.1 stress adaptor protein CpxP [Pantoea allii]
MRKLTAVVLASAMALSVASADAKDATTIDEMHHGGLPTGSMTQNPQSHMFDGIELTEEQRQQMRDLMQQ
ARHDRPVVHIDDIAALHELVTADQFNEAAIRQKAEVIARVQVEQQVEMARVQNQMFQLLTPAQQSTLQKNY
QRRLNELRQFSNLQSASSLQAVSSTSSNQ
>TGX88648.1 cell-envelope stress modulator CpxP [Pantoea agglomerans]
MRKLTAVVLASAMALSVASAGAKDATTIDEMHHGGLPTGSMTQNPQSHMFDGIELTEEQRQQMRDLMQQ
ARHERPVVHIDDIAALHELVTADQFNEAAIREKAEVIARVQVEQQVEMARVQNQMFQLLTPAQQSALQQNY
QRRLNELRQFSNLQSASSLQAVSSTSSNQ
>AUU28617.1 stress adaptor protein CpxP [Citrobacter freundii]
MGKVTAAVMASTLALSTFSHAAEVVTGDHWHLGEGSSQRSVQSHMFDGISLTEHQRQQMRDLMQQARHE
QPPVNVSEMETMHRLVTAEKFDESAVRAQAERMAQEQVARQVEIARIRNQMYRLLTPEQQAVLNEKHEQR
MVQLRDVAHWKKSSSLNLLSSSNSRSQ
